# Advances in Pig Genomics and Functional Gene Discovery

**DOI:** 10.1002/cfg.261

**Published:** 2003-04

**Authors:** Max F. Rothschild

**Affiliations:** 2255 Kildee Hall Department of Animal Science Center for Integrated Animal Genomics Iowa State University Ames IA 50011 USA

## Abstract

Advances in pig gene identification, mapping and functional analysis have continued to make rapid progress. The porcine genetic linkage map now has nearly 3000 loci, including several hundred genes, and is likely to expand considerably in the next few years, with many more genes and amplified fragment length polymorphism (AFLP) markers being added to the map. The physical genetic map is also growing rapidly and has over 3000 genes and markers. Several recent quantitative trait loci (QTL) scans and candidate gene analyses have identified important chromosomal regions and individual genes associated with traits of economic interest. The commercial pig industry is actively using this information and traditional performance information to improve pig production by marker-assisted selection (MAS). Research to study the co-expression of thousands of genes is now advancing and methods to combine these approaches to aid in gene discovery are under way. The pig's role in xenotransplantation and biomedical research makes the study of its genome important for the study of human disease. This review will briefly describe advances made, directions for future research and the implications for both the pig industry and human health.


**Comparative and Functional Genomics**
*Comp Funct Genom* 2003; **4**: 266–270.
Published online 1 April 2003 in Wiley InterScience (www.interscience.wiley.com). **DOI:** 10.1002/cfg.261

**Conference Review**

### Advances in pig genomics and functional gene discovery

Max F. Rothschild*
*Department of Animal Science, Center for Integrated Animal Genomics, Iowa State University Ames, IA 50011, USA*

**Correspondence to:
Max F. Rothschild, 2255 Kildee Hall, Department of Animal Science, Iowa State University, Ames, IA 50011, USA.
E-mail: mfrothsc@iastate.edu*

Received: 28 January 2003
Accepted: 28 January 2003

### Abstract

**Advances in pig gene identification, mapping and functional analysis have continued to make rapid progress. The porcine genetic linkage map now has nearly 3000 loci, including several hundred genes, and is likely to expand considerably in the next few years, with many more genes and amplified fragment length polymorphism (AFLP) markers being added to the map. The physical genetic map is also growing rapidly and has over 3000 genes and markers. Several recent quantitative trait loci (QTL) scans and candidate gene analyses have identified important chromosomal regions and individual genes associated with traits of economic interest. The commercial pig industry is actively using this information and traditional performance information to improve pig production by marker-assisted selection (MAS). Research to study the co-expression of thousands of genes is now advancing and methods to combine these approaches to aid in gene discovery are under way. The pig's role in xenotransplantation and biomedical research makes the study of its genome important for the study of human disease. This review will briefly describe advances made, directions for future research and the implications for both the pig industry and human health. Copyright © 2003 John Wiley & Sons, Ltd.**

**Keywords:** pig; gene identification; economic traits; functional genomics

### Introduction

The pig was among one of the first animals domesticated over 7000 years ago and pork is the major red meat consumed (43%) worldwide [23]. Furthermore, the pig has served as an important model system for human health and represents a significant future source of organs for transplantation. Efforts to unravel the pig genome began in the early 1990s with the development of the PiGMaP gene mapping project [1], which was initiated in Europe and was funded by the European Economic Community. PiGMaP involved 18 European labs and a total of 7 other labs from the USA, Japan and Australia. In the USA, the USDA launched two efforts. First, the USDA–ARS (US Department of Agriculture–Agricultural Research Service) began a sizeable gene mapping project [21] at the Meat Animal Research Center in Clay Center, Nebraska. Second, the National Animal Genome Research Program was developed under the direction of USDA–CSREES (Cooperative State Research Education and Extension Service) in 1993. This program was designed to provide a structure that included genome coordinators that would stimulate facilitation and collaboration of gene mapping in all species, including pigs. Scientists from state and private universities and federal labs cooperatively created a Swine Genome Technical Committee, which has met in recent years at the Plant and Animal Genome (PAG) Meetings. The US Pig Genome Coordinator activities, in concert with activities of the USDA–ARS and international gene mapping projects, such as PiGMaP and others, have allowed the status of the pig gene map to evolve more quickly and developments in functional genomics to advance rapidly in the last several years.

Copyright © 2003 John Wiley & Sons, Ltd.

### Gene mapping

New gene markers consisting of microsatellites, amplified fragment length polymorphisms (AFLPs) and single nucleotide polymorphisms (SNPs) continue to be identified and mapped and some integration of the maps continues to have taken place as quantitative trait maps are expanded. The largest single map contains about 1200 markers [21] but no new large-scale maps have been published recently. In total there are over 924 genes and 1641 markers in the database (**www.thearkdb.org/browser?species=pig**). There is a developing AFLP map with about 3000 AFLPs that is likely to be added to the PiGMaP linkage map some time in the future. Integration of the linkage, cytogenetic and physical maps is well under way with the development and use of chromosome painting [14], a somatic cell hybrid map [28] and a 7000 rad radiation hybrid (RH) panel (ImpRH) [30,15]. This RH map now contains nearly 3000 markers including microsatellites, and over 2000 new expressed sequence tags (ESTs), of which many are human orthologues and enable comparative mapping [20,24]. Continued use of these resources and development of an advanced 12 000 rad RH map are under way [29]. This will aid the rapidly developing comparative map, which will accelerate the identification of the genes explaining variation in traits of interest, either those identified by QTL studies or through direct approaches, such as gene association analyses.

### Database activities

Significant pig bioinformatics efforts have been initiated by the Roslin Institute, Scotland (**www.thearkdb.org**) and to a lesser extent in the USA (**www.genome.iastate.edu**) to support the pig genome efforts and display the gene maps [2]. PiGBASE, which can be reached through these sites, has several features, including pig gene mapping references with over 1093 citations in the database and gene maps with about 2565 loci. Last year there were over 2 million hits at these pig genome sites. Additional websites exist for the cytogenetic map of the pig (**http://www.toulouse.inra.fr/lgc/pig/cyto/cyto.htm**) and the RH panel map (**http://www.toulouse.inra.fr/lgc/pig/RH/Menu-chr.htm**). A comparative map is also on the web (**http://www.toulouse.inra.fr/lgc/pig/compare/compare.htm**). In addition, a new EST database (**http://pigest.genome.iastate.edu**) has been developed and should become a similarly useful resource. It is now accessible and contains over 98 988 pig EST entries and further development will continue. Other useful gene tools are available from the US pig genome website (**http://www.genome.iastate.edu**).

### QTL and candidate genes

Pork production requires efficient growth rate, reduced feed intake, carcass merit, meat quality and high levels of reproductive success and survivability. Using both commercial and exotic pig breeds, researchers have initiated experiments to identify quantitative trait loci (QTLs) affecting these traits. A large number of QTLs have been reported on nearly all chromosomes for growth, carcass and meat quality traits and several chromosomes for reproduction [3]. The QTLs affecting immune response traits and disease resistance are far less numerous. This is an area where gene expression approaches may be particularly valuable. Following discoveries of imprinted genes in other species, researchers have expanded their projects to find imprinted and origin-of-parent effects [10]. In particular, one such region on chromosome 2 has been intensively investigated [12] and IGF2 implicated in causing a major effect in muscle mass. The researchers cleverly employed a haplotype-sharing strategy analysis combined with marker-assisted segregation analysis to position the QTL within a 500 kb region. The causal quantitative trait nucleotide (QTN) was revealed after investigating over 180 SNPs and this work clearly points to the need for careful analysis of all gene regions and the proper animals and phenotypic information. Further evidence for imprinted regions and genes are likely to be found now that these approaches have been developed.

Candidate genes analyses have been employed to investigate a variety of traits. To date, significant associations have been demonstrated for candidate genes for litter size (ESR, PRLR, RBP4), growth (MC4R), meat quality (PRKAG3), disease resistance (FUT1, SLA, NRAMP) and coat color (KIT, MC1R) [3]. The commercial pig industry is actively using this gene marker information in

Copyright © 2003 John Wiley & Sons, Ltd. 

combination with traditional performance information to improve pig production by marker-assisted selection. Positional candidate gene analysis continues to be used to elucidate other known QTLs and has recently been useful in uncovering QTN mutations in PRKAG3 that affect pH and drip loss [6] and in CAST that affect tenderness [7]. It is likely that, as QTL experiments are expanded, additional positional candidates will be identified and the causative QTN discovered.

### Sequencing efforts

Research to date suggests that the porcine genome has a similar chromosomal organization ($2n = 38$, including meta- and acrocentric chromosomes), size ($3 \times 10^9$ bp), and complexity to the human genome. As with other species, researchers have generated ESTs from cDNA clones randomly picked from libraries from many tissues. These projects have varied in size and in the tissues used [8,17,19,26,27]. The largest of these types of projects published to date was sponsored by the USDA and reported the sequencing and initial analysis of 66 245 ESTs [11]. In addition, 21 499 sequences from reproductive tissue were produced by a consortium of several research groups [24]. At present, there are approximately 120 000 sequences in GenBank, and in the October 2002 TIGR release there were 17 350 clusters and 31 000 singletons. More deposits of 5000–10 000 EST sequences are expected soon. Most importantly, however, a major Sino-Danish effort to sequence the pig genome (**http://www.piggenome.dk/**) has resulted in approximately 700 000 EST sequences that are expected to be deposited in the database in the next 6–8 months. The data obtained by sequencing these large numbers of ESTs will continue to help assist comparative mapping efforts, candidate gene discovery and expression analysis.

Following the request of the NIH, a number of species have submitted requests to be considered for sequencing efforts. A 'White Paper' [22] was submitted to NHGRI recently that outlined the role the pig plays agriculturally, as well as a model for human biology. In addition to the efforts of the authors, the White Paper received solid backing from colleagues from several countries and from industry personnel from many companies and organizations. A cooperative project to develop a BAC map using the existing BAC library resources with approximately 35× coverage [22] has progressed nicely. It appears that the pig genome sequencing effort will receive a 'high priority ranking' but, despite these efforts, sufficient funding remains in question.

### Functional analysis

To better understand the physiological complexity of the pig transcriptome, expression and/or functional gene analysis needs to be undertaken. Initially such research was done using a limited number of genes and techniques, such as Northern analysis and differential display PCR [13,25]. Other approaches have included quantitative real-time PCR to determine mRNA levels for immune response and disease infection levels [9,18]. These approaches, while quite useful, have proved to be limited in the numbers of genes that can be considered. Other approaches have included use of limited numbers of cDNAs on macroarrays [31]. Given the initial lack of development of large-scale cDNA arrays for the pig, human arrays have been tested and used [13,16]. Experiments with such materials have proved initially valuable, as reproducibility was generally high and results were reasonable. However, the recent advent of large numbers of pig ESTs has allowed for large-scale expression analysis using porcine materials only. Pomp and colleagues [4,5] have used cDNA derived from ovary and follicular RNA from animals from either an index line selected for higher litter size or a control line, and co-hybridized them with 4600 follicle-derived probes to study gene expression patterns related to reproductive efficiency. Other projects exist including two large-scale efforts in Europe. The first European Community-supported project is called PathoCHIP (**http://www.pathochipproject.com**) and uses spotted cDNA arrays for disease organism and immune response genes, while the second, called QualityPorkGENES (**www.qualityporkgenes.com**) looks at the co-expression of genes related to meat quality. Cooperative efforts by the US Pig Genome Coordinator and US and International researchers have now been directed to developing a first stage cDNA or oligo spotted array for the pig genome and human biomedical community. It is expected that such an array will be commercially

Copyright © 2003 John Wiley & Sons, Ltd.

available in mid-2003. This array and others to be developed later will advance functional analysis in the pig.

### Conclusions

Understanding the complexity of the pig genome for both agricultural purposes and for its importance to human biomedical concerns remains a significant challenge. In the past decade, large-scale gene and trait identification and mapping have taken place and a number of gene tests to improve pork production are in use in the pig industry. Sequencing and expression analysis have been initiated and offer new avenues to understand the biological complexity of the pig. No longer does the pig genomics community rely solely on developments from other organisms, such as the human and the mouse. This sentiment was also shared by Nobel laureate Dr Sydney Brenner at this year's PAG meeting, where Dr Brenner stated, 'more serious people work on important things', expressing his belief that scientists should now focus on their primary species and not model systems. The state of the art of pig gene discovery and functional genomics clearly demonstrates such commitment and progress.

### Acknowledgements

This is a journal paper of the Iowa Agriculture and Home Economics Experiment Station, Ames, IA, USA, Project No. 3148, and was supported in part by Hatch Act and State of Iowa funds and funding from NRSP-8 and the USDA/CSREES Pig Genome Coordination program. The efforts of all scientists involved in the pig gene mapping and genomics programs worldwide are gratefully acknowledged, including efforts by Alan Archibald, Andy Law and colleagues, Roslin Institute, and Zhiliang Hu to support database activities. Comments and information supplied by H. Cheng, C. Ernst, M. Georges, G. Plastow, D. Pomp and C. Tuggle are appreciated.

### References

1. Archibald A, Brown J, Couperwhite S, *et al*. 1995. The PiGMaP consortium linkage map of the pig (*Sus scrofa*). *Mamm Genome* **6**: 157–175.
2. Archibald A, Law A. 2003. Informatics for pig genome research. *Proceedings of the Plant and Animal Genome XI Meetings,* San Diego, CA. **http://www.intl-pag.org/11/abstracts/W52_W329_XI.html**.
3. Bidanel JP, Rothschild MF. 2002. Current status of quantitative trait locus mapping in pigs. *Pig News Inform* **23**: 39N–53N.
4. Caetano AR, Johnson RK, Pomp D. 2002. RNA expression profiling of ovarian follicle development on swine lines selected for increased ovulation rate. Communication No. 08–07. *Proceedings of the 7th World Congress on Genetics as Applied to Livestock Production*, August 19–23, Montpellier, France.
5. Caetano AR, Johnson RK, Pomp D 2003. Using cDNA microarrays to study ovarian follicle development in pigs selected for increased ovulation rate. *Proceedings of the Plant and Animal Genome XI Meetings,* San Diego, CA. **http://www.intl-pag.org/11/abstracts/W52_W328_XI.html**.
6. Ciobanu DC, Bastiaansen J, Malek M, *et al*. 2001. Evidence for new alleles in the protein kinase AMP-activated, subunit gene associated with low glycogen content in pig skeletal muscle and improved meat quality. *Genetics* **159**: 1151–1162.
7. Ciobanu DS, Lonergan SM, Bastiaansen JWM, *et al*. 2002. Evidence for new alleles in the calpastatin gene associated with meat quality traits. Communication No. 11–10. *Proceedings of the 7th World Congress on Genetics as Applied to Livestock Production*, August 19–23, Montpellier, France.
8. Davoli R, Fontanesi L, Zambonelli P, *et al*. 2002. Isolation of porcine expressed sequence tags for the construction of a first genomic transcript map of the skeletal muscle in pig. *Anim Genet* **33**: 3–18.
9. Dawson H, Nishi S, Beshah E, *et al*. 2003. Use of real-time assays of immune gene expression to assess the genetic basis of disease resistance. *Proceedings of the Plant and Animal Genome XI Meetings,* San Diego, CA. **http://www.intl-pag.org/11/abstracts/W52_W330_XI.html**.
10. De Koning DJ, Rattink AP, Harlizius B, *et al*. 2000. Genome-wide scan for body composition in pigs reveals important role of imprinting. *Proc Natl Acad Sci USA* **97**: 7947–7950.
11. Fahrenkrug SC, Smith TP, Freking BA, *et al*. 2002. Porcine gene discovery by normalized cDNA-library sequencing and EST cluster assembly. *Mamm Genome* **13**: 475–478.
12. Georges M, Andersson G, Braunschweig M, *et al*. 2003. Genetic dissection of an imprinted QTL mapping to prosimal SSC2. *Proceedings of the Plant and Animal Genome XI Meetings,* San Diego, CA. **http://www.intl-pag.org/11/abstracts/W52_W327_XI.html**.
13. Gladney CD, Bertani GR, Johnson RK, Pomp D. 2003. Evaluation of gene expression in pigs selected for enhanced reproduction using differential display PCR and microarray. I. Ovarian follicles. *J Anim Sci* (submitted).
14. Goureau A, Yerle M, Schmitz A, *et al*. 1996. Human and porcine correspondence of chromosome segments using bidirectional chromosome painting. *Genomics* **36**: 252–262.
15. Hawken RJ, Murtaugh J, Flickinger GH. 1999. A first-generation porcine whole-genome radiation hybrid map. *Mamm Genome* **10**: 824–830.
16. Moody DE, Zou Z, McIntyre L. 2002. Cross-species hybridization of pig RNA to human nylon microarrays. *BMC Genom* **3**: 27.
17. Nobis WP, Coussens PM 2003. Development of porcine brain EST and cDNA library and microarray resources. *Proceedings of the Plant and Animal Genome XI Meetings,* San Diego, CA. **http://www.intl-pag.org/11/abstracts/P7a_P749_XI.html**.

Copyright © 2003 John Wiley & Sons, Ltd. 

18. Okomo-Adhiambo MA, Beattie CW, Rink A 2003. Relative quantification of the differential expression of apoptosis genes in an *in vitro* porcine *Toxoplasma gondii* infection using real-time RTPCR. *Proceedings of the Plant and Animal Genome XI Meetings,* San Diego, CA. **http://www.intl-pag.org/11/abstracts/P7c_P840_XI.html**.
19. Rink A, Santschi EM, Beattie CW. 2002a. Normalized cDNA libraries from a porcine model of orthopedic implant-associated infection. *Mamm Genome* **13**: 198–205.
20. Rink A, Santschi EM, Eyer KM, *et al.* 2002b. A first-generation EST RH comparative map of the porcine and human genome. *Mamm Genome* **13**: 578–587.
21. Rohrer GA, Alexander LJ, Hu Z, *et al.* 1996. A comprehensive map of the porcine genome. *Genome Res* **6**: 371–391.
22. Rohrer G, Beever JE, Rothschild MF, *et al.* 2002. Porcine Genomic Sequencing Initiative. National Human Genome Research Institute (submitted). **http://www.genome.iastate.edu/newsletter/PigWhitePaper.html**.
23. Rothschild MF, Ruvinsky A. 1998. *Genetics of the Pig*. CABI Press: Wallingford, UK; 622.
24. Tuggle CK, Green JA, Fitzsimmons C, *et al.* 2003. EST-based gene discovery in pig: virtual expression patterns and comparative mapping to human. *Mamm Genome* (submitted).
25. Wilson ME, Fahrenkrug SC, Smith TP, *et al.* 2002. Differential expression of cyclooxygenase-2 around the time of elongation in the pig conceptus. *Anim Reprod Sci* **71**: 229–237.
26. Winterø AK, Fredholm M, Davies W. 1996. Evaluation and characterization of a porcine small intestine cDNA library: analysis of 839 clones. *Mamm Genome* **7**: 509–517.
27. Yao J, Coussens PM, Saama P, *et al.* 2002. Generation of expressed sequence tags from a normalized porcine skeletal muscle cDNA library. *Anim Biotechnol* **13**: 211–202.
28. Yerle M, Echard G, Robic A, *et al.* 1996. A somatic cell hybrid panel for pig regional gene mapping characterized by molecular cytogenetics. *Cytogenet Cell Genet* **73**: 194–202.
29. Yerle M, Pinton P, Delcros C, *et al.* 2002. Generation of a 12 000 rad radiation hybrid panel for fine mapping in pigs. *Cytogenet Genome Res* **97**: 219–228.
30. Yerle M, Pinton P, Robic A, *et al.* 1998. Construction of a whole-genome radiation hybrid panel for high-resolution gene mapping in pigs. *Cytogenet Cell Genet* **82**: 182–188.
31. Zhao S, Nettleton D, Liu W, *et al.* 2003. cDNA macroarray analyses of differential gene expression in porcine fetal and postnatal muscle. RTPCR. *Proceedings of the Plant and Animal Genome XI Meetings,* San Diego, CA. **http://www.intl-pag.org/11/abstracts/P5m_P623_XI.html**.

Copyright © 2003 John Wiley & Sons, Ltd. 
